# The Predictive Value of 2D Myocardial Strain for Epirubicin-Induced Cardiotoxicity

**DOI:** 10.1155/2020/5706561

**Published:** 2020-11-30

**Authors:** Ichrak Ben Abdallah, Sonia Ben Nasr, Chadia Chourabi, Marouane Boukhris, Israa Ben Abdallah, Aref Zribi, Sana Fendri, Mehdi Balti, Wafa Fehri, Nesrine Chraiet, Abderrazek Haddaoui

**Affiliations:** ^1^Department of Medical Oncology, Military Hospital of Tunis, Université de Tunis El Manar, Faculté de Médecine de Tunis, Tunis 1007, Tunisia; ^2^Department of Cardiology, Military Hospital of Tunis Université de Tunis El Manar, Faculté de Médecine de Tunis, Tunis 1007, Tunisia; ^3^Division of Cardiology, Centre Hospitalier de l'université de Montréal, Montreal, Québec, Canada; ^4^Department of Business Analytics, Tunis Business School, El Mourouj, Tunisia; ^5^Department of Medical Oncology, Université de Tunis El Manar, Faculté de Médecine de Tunis, Tunis 1007, Tunisia

## Abstract

**Introduction:**

Although epirubicin has significantly improved outcome in breast cancer (BC) patients, it is responsible for myocardial dysfunction that affects patients' quality of life. The use of 2D global longitudinal strain (GLS) has been reported to detect early myocardial dysfunction. The aim of this study was to evaluate how GLS changes can predict cardiotoxicity.

**Methods:**

We conducted a prospective study from March 2018 to March 2020 on 66 patients with no cardiovascular risk factors, who presented with BC and received epirubicin. We measured left ventricular ejection fraction (LVEF) and GLS before chemotherapy, at three months (T3), and at 12 months (T12) from the last epirubicin infusion. Chemotherapy-Related-Cardiac-Dysfunction (CTRCD) was defined as a decrease of 10% in LVEF to a value below 53% according to ASE and EACI 2014 expert consensus.

**Results:**

The mean age at diagnosis was 47 ± 9 years old. At baseline, median LVEF was 70% and median GLS was −21%. Shortly after chemotherapy completion, two patients presented with symptomatic heart failure while asymptomatic CTRCD was revealed in three other patients at T12. Three months after the last epirubicin infusion, median LVEF was 65%, median GLS was −19%, and median GLS variation was 5%. However, in patients who presented with subsequent CTRCD, median GLS at T3 was −16% and median GLS variation was **19%** (*p*=0.002 and *p* < 0.001, respectively, when compared to patients who did not develop cardiotoxicity). Persistent GLS decrease at T3 was an independent predictor of CTRCD at T12. Age and left-sided thoracic irradiation did not increase the risk of cardiotoxicity in our study while the cumulative dose of epirubicin significantly affected cardiologic findings (*p*=0.001).

**Conclusion:**

This was the first North African study that assesses the value of measuring GLS to early detect cardiotoxicity. Patients whose GLS remained decreased after 3 months from anthracyclines-base chemotherapy had an increased risk for developing subsequent CTRCD. Further studies with larger sample size are warranted to identify the best cardioprotective molecules to be initiated in these patients before LVEF declines.

## 1. Introduction

Epirubicin, a commonly used chemotherapeutic agent in the treatment of breast cancer, has significantly improved the survival in these patients. Epirubicin-based regimens are considered as a standard of care in the adjuvant, neoadjuvant, and metastatic settings [1]. However, the wide use of this drug has led to increased awareness of its side effects including its potential cardiac toxicity mainly to the myocardium, which can lead to heart failure (HF) and death [2].

Although left ventricular ejection fraction (LVEF) assessment during and after treatment is considered as a predictive tool of symptomatic HF, decreased LVEF may occur late after treatment, justifying the need for more sensitive predictive tools for LV dysfunction [3, 4]. Indeed, recent studies have demonstrated the predictive value of global longitudinal strain (GLS) in the detection of subclinical LV dysfunction in patients treated for cancer [3, 4].

To the best of our knowledge, there has been no published data evaluating the use of GLS for the detection of chemotherapy-related cardiac dysfunction (CTRCD) in African cancer patients.

The aim of our study was to assess the role of GLS to early detect CTRCD in Tunisian breast cancer patients with no cardiovascular risk factors.

## 2. Methods

### 2.1. Study Population

We conducted a prospective study from March 2018 to March 2020 including 66 breast cancer patients treated with epirubicin-based regimens. Patients presenting with the following comorbidities were excluded: diabetes, hypertension, coronary artery disease, thyroid dysfunction, dyslipidemia, preexisting cardiomyopathy and/or valvular disease, and autoimmune diseases. Active smokers were also excluded. Finally, patients who received further Trastuzumab treatment were beyond the scope of the current study.

### 2.2. Epirubicin Treatment

The type of epirubicin-based regimen and the number of courses were determined according to the patient's risk of recurrence on a collegial decision. The main regimens adopted were as follows:

- FEC100: Fluorouracil 500 mg/m^2^ IV Day 1
- Epirubicin 100 mg/m^2^ IV Day 1
- Cyclophosphamide 500 mg/m^2^ IV Day 1
- EC: Epirubicin 90 mg/m^2^ IV Day 1
- Cyclophosphamide 600 mg/m^2^ IV Day 1

The time interval between the two courses was 21 days

### 2.3. Transthoracic Echocardiogram and GLS Assessment

Transthoracic echocardiograms were performed using the Vivid 7 or E9 by the same cardiologist expert in cardiac imaging. GLS was calculated using the EchoPAC™ software. All patients had echocardiograms at baseline, after three months (T3), and after 12 months (T12) from the last epirubicin infusion. CTRCD was defined as a decrease in the LVEF greater than 10 percentage points, to a value below 53% according to the 2014 experts' consensus of the American Society of Echocardiography (ASE) and the European Association of Cardiovascular Imaging (EACI) [3]. To test intrainvestigator variability, ten randomly selected patients had a second echocardiography examination performed by the same investigator within one week from the first examination. The degree of agreement was determined using the Kappa coefficient.

### 2.4. Statistical Analysis

Data analysis was processed using SPSS version 21 (IBM Corporation, Armonk, New York).

First, we analyzed patients' and disease baseline characteristics. Continuous variables were presented as means ± standard deviation for the normally distributed variables and as medians (with range intervals) for other distributions. Nominal variables were expressed as percentages. To compare percentages, we used the Chi2 test and Fisher's exact test when needed. Normally distributed continuous variables were compared using the *T*-test. For the other variables (mainly echocardiographic data), we used the Mann–Whitney *U* test for univariate analysis. Multivariate analysis was performed by a logistic regression model to assess the potential independent predictors of cardiotoxicity. *p* value <0.05 was considered statistically significant. Finally, ROC curve analysis was performed to determine the cut-off value for parameters that were significantly associated with the occurrence of CTRCD.

## 3. Results

### 3.1. Patients' Characteristics

Sixty-six newly diagnosed patients with breast cancer who were eligible for epirubicin-based regimens in adjuvant or metastatic settings were enrolled in the study. Patients' characteristics and disease features are summarized in Table 1.

### 3.2. Chemotherapy Regimens

The median dose of epirubicin was 350 mg/m^2^ (200–900). Patients with the metastatic disease received a median dose of 600 mg/m^2^ (300–900) while patients conducting adjuvant therapy received a median dose of 300 mg/m^2^ (200–600). Adjuvant chemotherapy regimens used in the study are summarized in Table 2. In the metastatic setting, all patients received FEC100 exclusively.

### 3.3. Echocardiography Parameters and GLS Assessment

Among the 66 patients included, five experienced cardiac toxicity (7.6%): two patients (A and B) showed symptomatic heart failure after the completion of chemotherapy, while in the three remaining patients, CTRCD was asymptomatic at the 12-month control. The clinical presentation of patients A and B is summarized in Supplement 1. We also report that one patient (C) presented with poorly tolerated palpitations without myocardial strain impairment, requiring withdrawal of anthracyclines (Supplement 1).

Univariate analysis was conducted to assess clinical and baseline echocardiographic data of all patients who did or did not present with CTRCD during follow-up (Table 3). Of note, as patients A and B already presented with CTRCD before T3, they were excluded from further analysis.  Table 4 summarizes univariate analysis for potential echocardiographic predictors of CTRCD at T12 for patients who did not experience CTRCD at T3. Only GLS and GLS variation at T3 were significantly associated with increased risk of CTRCD at T12.

When performing multivariate analysis with logistic regression model (Table 5), only GLS variation at T3 was an independent predictive factor for CTRCD at T12 (*p*=0.008). A certain trend toward significance was noted with the GLS value at T3 (*p*=0.08). The accuracy of the model was 98%.

### 3.4. Predictive Value of GLS

**Figure 1:**
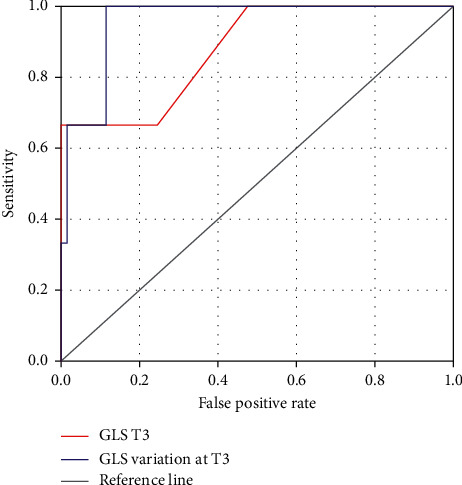
Receiver operating characteristic curves for parameters predictive of CTRCD.

Figure 1 shows the predictive value of echocardiographic parameters for CTRCD with their sensitivity and specificity:

- T3 GLS variation curve had the highest predictive value for CTRCD (AUC [0.95 (95% CI of 0.88–1)]. The cut-off value was 9% (sensitivity = 100%; specificity = 88%; odds ratio = 8.7).
- The AUC for GLS at 3 months was 0.88 (95%CI of 0.67–1). The optimal cut-off point was −16.4% (sensitivity = 67; specificity = 66%; odds ratio = 48).

Intraobserver reproducibility was assessed and Kappa coefficient was 0.81 for LVEF and 0.89 for GLS indicating a strong level of agreement for both LVEF and GLS.

**Figure 2:**
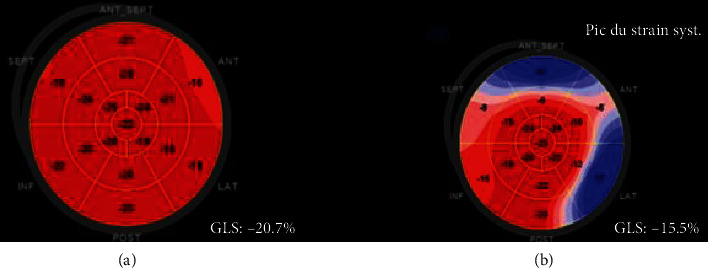
Longitudinal strain bull's eyes showing normal GLS before chemotherapy (a) and reduced GLS predominant in basal segments after chemotherapy (b).

Figures 2 illustrates normal strain (before chemotherapy) and decreased strain (after chemotherapy) in one patient.

## 4. Discussion

The main findings of the current study can be summarized as follows: in patients with breast cancer with no comorbidities who received epirubicin-based regimens, CTRCD occurred in 7.6% of cases and was symptomatic in 2 patients (3%) with early onset of symptoms. A GLS that remains decreased three months after the end of anthracyclines was an independent predictor for CTRCD at T12 with an optimal cut-off value of 9% in GLS variation (100% sensitivity, 88% specificity).

The study population was relatively young as the median age at diagnosis was 46 years old and 71% of the patients were premenopausal. This is mainly due to two reasons. The first reason is the patients' selection criteria. Indeed, excluded patients with cardiovascular risk factors are generally those older.

Second, previous epidemiologic studies reported that breast cancer in the Tunisian population is more likely to occur in younger adults [5–7]. In fact, frequency peak usually occurs between 40 and 50 years with a mean age of 51, unlike European countries where the disease predominantly affects women aged ≥60 [8, 9].

Symptomatic heart failure occurred within one month after chemotherapy in two patients. What was unusual is the rapidity of the onset of heart failure symptoms observed in these patients. Some previous studies reported such an early onset secondary to anthracyclines, mainly with doxorubicin [10]. This highlights the need for cardiac monitoring throughout chemotherapy and not only after the completion of treatment as some patients may be more susceptible to develop earlier heart damage due to anthracyclines [10]. The prevalence of symptomatic heart failure previously reported in patients receiving epirubicin-based regimens ranges from 1 to 8% for small doses of epirubicin not exceeding 500 mg/m^2^ [10–12]. In patients receiving higher cumulative doses (≥900 mg/m^2^), symptomatic CTRCD prevalence may exceed 5 to 10% [11]. The small number of patients presenting with symptomatic heart failure in our study (3%) is mainly due to our patients' selection criteria. As a matter of fact, in studies considering patients with limited baseline left ventricular ejection fraction ranging from 50 to 59% and treated with anthracyclines, the incidence of symptomatic heart failure was 8% [10].

Patients presenting with asymptomatic CTRCD represented 4.6% of our cohort. The incidence of cardiotoxicity reported in previous studies varies according to the different used definitions [1]. It ranged from 1 to 11% for small doses of epirubicin and may exceed 20% for more important cumulative doses and after longer follow-up [13, 14].

Our findings also highlighted that CTRCD is a dose-dependent event as patients presenting with cardiotoxicity received a median dose of 600 mg/m^2^ versus 300 mg/m^2^ in nonaffected patients and the difference was statistically significant (*p*=0.001). The role of cumulative dose of anthracyclines in the process of cardiotoxicity has already been established in previous reports [15, 16]. The commonly agreed upper limit for epirubicin in routine practice is 950 mg/m^2^; exceeding this limit exposes the patient to a 15% higher risk of cardiotoxicity [17]. However, according to the 2017 American society of clinical oncology clinical practice guidelines on the prevention and monitoring of cardiac dysfunction in survivors of adult cancers, patients who receive a cumulative dose of epirubicin ≥600 mg/m^2^ are considered to be at increased risk for developing cardiac dysfunction [15, 18]. Nonetheless, it is important to realize that there is likely no “safe” anthracycline dose where cardiotoxicity will not occur and that the cardiotoxicity process may begin with the first dose of anthracycline [15, 17]. This is mainly related to interindividual susceptibility to these drugs which is regulated by genetic predisposition and single nucleotide polymorphisms (SNPs) [15, 19, 20]. We recall that the main mechanism of action of anthracyclines is intercalation between DNA strands resulting in inhibition of DNA and RNA synthesis [21] and generation of free radicals, which leads to apoptosis [22].

The main purpose of our study was to assess the added value of echocardiographic parameters for the detection of later CTRCD. Only GLS and GLS variation at 3 months were significantly associated with cardiotoxicity with GLS variation being an independent predictive factor in the regression model. Conversely, LVEF at 3 months was still in the normal range in these patients and was not predictive of cardiotoxicity at T12 (*p*=0.577). However, it is important to realize that only 3 patients presented with subsequent CTRCD at T12 which reduces the statistical power of the current study. Studies with larger sample sizes are needed to further elucidate the prognostic value of GLS measures.

The ability of LVEF assessment during and after treatment to identify CTRCD and prevent subsequent heart failure remains controversial and problematic [3]. First, LVEF assesses the global and segmental left ventricular function depending on geometrical assumptions and functional factors which demonstrates how LVEF can remain preserved (with increased LV wall thickness or reduced LV diameter) despite decreased measures of strain [23]. Second, detecting a decrease in LVEF after anthracyclines may be irreversible and too late for treatment, which cannot allow taking any preventive measures. We recall that although the two patients A and B who presented with symptomatic heart failure received ACEIs and beta-blockers, their ejection fraction remained impaired during follow-up. This suggests that more sensitive parameters are needed and that speckle-tracking use between chemotherapy cycles would be an alternative option [3, 24].

Considering that endocardial longitudinal fibers are claimed to be the most susceptible to ischemic injury and the first to suffer from anthracyclines toxicity, GLS, which is a measure of the active shortening of the LV in the longitudinal direction, seems to be a sensitive tool for detection of subclinical myocardial dysfunction [25, 26]. Of note, the reason why patients with comorbidities were excluded from our study is that these patients may present a worsening of their myocardial strain resulting from their preexisting illness as suggested by Mousavi et al. who could not subsequently conclude whether the strain alteration was due to anthracyclines or to a worsening of the patients' initial cardiovascular condition [10].

The threshold value for GLS decrease noted in our findings was 9% (Se = 100, SP = 88%) versus 15% in the 2014 expert consensus [3]. Baratta et al. also reported that a 15% decrease in GLS had 86% sensitivity and 86% specificity for prediction of later CTRCD while Negishi et al. reported that an 11% reduction in global GLS had 65% sensitivity and 95% specificity [27, 28]. This slight difference between our findings and those previously reported is more likely to be the result of patients' selection criteria which suggests that threshold values should be adjusted to patients' cardiovascular risk factors [28, 29].

Similar to GLS, in the absence of clear consensus, most of the previous studies reported predictive values of GLS variation ranging from −14% to −19% [3, 28, 29].

Finally, radiotherapy was not associated with an increased risk of CTRCD at 12 months. A systematic review on the long-term risk of heart failure and myocardial dysfunction after thoracic radiotherapy concluded that although patients receiving thoracic radiotherapy presented with a decrease in their myocardial strain, their ejection fraction remained often normal [12]. This is mainly due to the radioresistant character of the myocardium and that the mechanism of cardiotoxicity of radiotherapy is radiation-induced coronaropathy and pericarditis [30].

### 4.1. Study Limitations and Strength

The main limitation of the current study is the small sample size of the cohort with regard to the rarity of the investigated event (CTRCD). This was mainly due to patients' selection criteria as many patients aged over 50 years old who were diagnosed with breast cancer had cardiovascular risk factors (such as diabetes and hypertension) were excluded. In addition, only a one-year follow-up made some difficulties to draw clear conclusions on the cardiovascular outcome of these patients.

Nonetheless, to the best of our knowledge, this was the first North African study to assess the added value of GLS measurement as a predictive tool for cardiotoxicity in patients treated with epirubicin. We highlight the importance of patients' selection criteria adopted in this study (exclusion of patients with comorbidities) to minimize confounding factors for GLS decrease. We also ensured to minimize measurement bias by avoiding interobserver variability (same investigator for all echocardiograms) and by the prospective nature of the study.

## 5. Conclusion

The development of breast cancer therapies significantly improved outcomes in patients and shifted the care pattern from cancer survival to cancer “survivorship.” The use of LVEF for the screening of cardiotoxicity in cancer survivors may be too late to allow intervention and to introduce preventive measures. The global longitudinal systolic strain of the left ventricle was found to be an accurate tool for detection of subclinical myocardial dysfunction before LVEF declines, thus allowing starting cardioprotective treatment before heart failure occurs. Patients who had a persistent decrease in GLS three months after the end of chemotherapy had an independent risk of developing subsequent CTRCD. Further larger multicentric studies evaluating the best cardioprotective molecules to be initiated in these patients should be conducted to elaborate on new and common guidelines.

## Figures and Tables

**Table 1:** Patients' characteristics and disease features.

Age, years, mean ± SD	47 ± 9
Postmenopausal women, *n* (%)	19 (29%)
TNM stage, *n* (%)	Stage I 14 (21%)
	Stage II 22 (33%)
	Stage III 19 (29%)
	Stage IV 11 (17%)
Molecular profile, *n* (%)	Luminal A 9 (14%)
	Luminal B 47 (71%)
	Triple negative 10 (15%)
Cancer side, *n* (%)	Right sided 35 (53%)
	Left sided 31 (47%)
Locoregional radiotherapy, *n* (%)	56 (85%)
Locoregional therapy for left-sided breast cancer, *n* (%)	27 (41%)

**Table 2:** Adjuvant chemotherapy regimens used in our study.

Regimen	Percentage
FEC100 +Paclitaxel, *n* (%)	**48 (72%)**
FEC100 +Docetaxel, *n* (%)	**5 (8%)**
FEC100, *n* (%)	**10 (15%)**
EC *n* (%)	**3 (5%)**

**Table 3:** Univariate analysis of baseline parameters and clinical features of patients who did or did not present with cardiotoxicity during follow-up.

	All patients (*n* = 66)	CTRCD+ (*n* = 5)	CTRCD-(*n* = 61)	*p* value
Age, years, median [ranges]	**46** [27–70]	**45** [42–47]	**47** [27–70]	0.125
Epirubicin cumulative dose (mg/m^2^), median [ranges]	**350** [200–900]	**600** [400–900]	**300** [200–900]	0.001
LVEF T0 (%), median [ranges]	**70** [58–75]	**67** [62–75]	70 [58–75]	0.670
GLS T0 (%), median [ranges]	−**21** [−25, −18]	−**21** [−22, −19]	−**21** [−25, −18]	0.597
Adjuvant LRR for left-sided BC, *n* (%)	**27** (41%)	**1** (20%)	**26** (42%)	0.323
Cancer stage:				0.098
Early disease, *n* (%)	**56** (85%)	**2** (40%)		
Metastatic disease, *n* (%)	**10** (15%)	**3** (60%)		

CTRCD+: patients who presented with cardiotoxicity during follow-up. T0: at baseline. CTRCD-: patients who did not present with cardiotoxicity during follow-up. T3: at three months. GLS: global longitudinal strain. LVEF: left ventricular ejection fraction. LRR: locoregional radiotherapy. BC: breast cancer. Ps: as patients A and B already presented with CTRCD before T3, they were excluded from further analysis.

**Table 4:** Univariate analysis of echocardiographic predictors of cardiotoxicity at 12 months.

	All patients (*n* = 64)	C+ (*n* = 3)	C− (*n* = 61)	*p*-value
Median LVEF T3	**65%** [54–76]	**63%** [54–68]	**61%** [54–76]	**0.408**
Median GLS T3	−**19.5%** [−24, −15]	−**16%** [−19, −15]	−**20%** [−24, −16.8]	**0.022**
Median GLS T3 variation	**4.8%** [0–28.5]	**15.7%** [9–28.5]	**4.7%** [4–23.5]	**0.008**

CTRCD+: patients who presented with cardiotoxicity at T12 consultation. CTRCD-: patients who did not present with cardiotoxicity during follow-up. T3: at three months. GLS: global longitudinal strain. LVEF: left ventricular ejection fraction.

**Table 5:** Logistic regression model for cardiotoxicity predictors.

	*p* value
LVEF T3 (%)	0.577
GLS T3 (%)	0.08
GLS variation T3 (%)	0.008
Cumulative epirubicin dose (mg/m^2^)	0.280
Cancer stage (early vs. metastatic)	0.803
Age	0.483

T3: at three months. T12: at 12 months. GLS: global longitudinal strain. LVEF: left ventricular ejection fraction.

## Data Availability

Data are available from the corresponding author upon reasonable request.

## Supplementary Materials

Supplementary Materials The clinical presentation of patients A, B, and C is summarized in Supplement 1.
