# Supplementary material for: The Predictive Value of 2D Myocardial Strain for Epirubicin-Induced Cardiotoxicity
Source: J Oncol. 2020 Nov 30;2020:5706561. doi: 10.1155/2020/5706561 (PMC7723482; doi:10.1155/2020/5706561)
Supplement: Supplementary Materials — The clinical presentation of patients A, B, and C is summarized in Supplement 1. [file 5706561.f1.docx]

| **Patient** | **Clinical presentation** |
| --- | --- |
| **Patient A** | **P**  A 43-year-old patient was diagnosed in 2018 with stage IV triple negative breast cancer. She received a cumulative dose of epirubicin of 600mg/m² with complete response. One month after the last infusion of epirubicin, she was referred to the cardiology department for progressive dyspnea. Her complete blood count was normal. Physical examination revealed pulmonary crackles and Chest X-ray showed Kerley B lines with interstitial edema concluding to pulmonary congestion. Her LVEF was 50% vs 76% at baseline, her GLS was -13% vs -22%, and her electrocardiogram was within normal limits. An Angiotensin Converting Enzyme inhibitor (ACEI) was introduced (Ramipril 10mg/day) with furosemide IV 125mg/day during hospitalization. Three months’ and 12 months’ controls revealed LVEF of 52% and 50% with improved symptoms on Ramipril 10mg/day. |
| **Patient B** | A 46-year-old patient received 900 mg/m² cumulative dose of epirubicin for stage IV breast cancer. She presented to our emergency department for stage III NYHA dyspnea two weeks after the last infusion of epirubicin. Examination revealed tachycardia and crackles at both lung bases revealing acute pulmonary edema. Transthoracic echocardiogram showed decrease in LVEF to 25% vs 75% at baseline. Her GLS was -12% vs -20% at baseline. First-line therapy for heart failure with ACEI (Ramipril 10mg/day), spironolactone (75mg/day) and furosemide IV was immediately started. After relief of the pulmonary edema, bisprolol (2.5mg/day) was initiated at a dose of 2.5mg/day. The maximum tolerated dose in this patient was 5mg/day. Her three months’ echocardiogram did not show any improvement (LVEF =25% and GLS=-11%). However at 12 months, her dyspnea was partially improved. Her LVEF was 40% and her GLS was -14.5%. She was on endocrine therapy with anastrozole and had stable disease. |
| **Patient C** | A33-year-old female was diagnosed with stage III breast cancer and was suggested to receive 4 courses of FEC100 regimen in the adjuvant setting. She received only two FEC100 courses because she presented poorly tolerated palpitations following each epirubicin infusion with documented premature ventricular beats that had been spontaneously resolved within 24 hours. Epirubicin-based regimens were definitively contraindicated in this patient. Follow-up did not reveal any LVEF impairment (LVEF =65% versus 66% at baseline). At the three months’ control we did not observe any other patient presenting CTRCD. At T12, three other patients presented CTRCD with no apparent symptoms. |
